# Glutaredoxin 1 mediates the protective effect of steady laminar flow on endothelial cells against oxidative stress-induced apoptosis via inhibiting Bim

**DOI:** 10.1038/s41598-017-15672-3

**Published:** 2017-11-14

**Authors:** Yao Li, Meng Ren, Xiaoqun Wang, Xingxing Cui, Hongmei Zhao, Chuanrong Zhao, Jing Zhou, Yanan Guo, Yi Hu, Chen Yan, Bradford Berk, Jing Wang

**Affiliations:** 1 State Key Laboratory of Medical Molecular Biology, Department of Pathophysiology, Institute of Basic Medical Sciences, Chinese Academy of Medical Sciences, School of Basic Medicine, Peking Union Medical College, Beijing, 100005 China; 2 Department of Cardiology, Ruijin Hospital, Shanghai Jiao-Tong University school of medicine, Shanghai, 200025 China; 3 Department of Physiology and Pathophysiology, School of Basic Medical Sciences, Peking University, Beijing, China; Key Laboratory of Molecular Cardiovascular Science, Ministry of Education, Beijing, China; 4 Aab Cardiovascular Research Institute, School of Medicine and Dentistry, University of Rochester, Rochester, NY 14642 USA; 5 CAS Key Laboratory for Biomedical Effects of Nanomaterials and Nanosafety, Multi-disciplinary Research Division, Institute of High Energy Physics, Chinese Academy of Sciences (CAS), Beijing, 100049 China

## Abstract

Endothelial cell apoptosis induced by oxidative stress is an early event in the development of atherosclerosis. Several antioxidant enzymes which can cope with oxidative stress are up-regulated by the anti-atherogenic laminar blood flow often seen in straight or unbranched regions of blood vessels. However, the molecular mechanism responsible for flow-induced beneficial effects is incompletely understood. Here we report the role of glutaredoxin 1 (Grx1), an antioxidant enzyme, in flow-mediated protective effect in endothelial cells. Specifically, we found that Grx1 is markedly up-regulated by the steady laminar flow. Increasing Grx1 reduces the pro-apoptotic protein Bim expression through regulating Akt-FoxO1 signaling and also attenuates H_2_O_2_-induced Bim activation via inhibiting JNK phosphorylation, subsequently preventing the apoptosis of endothelial cells. Grx1 knockdown abolishes the inhibitory effect of steady laminar flow on Bim. The inhibitory effect of Grx1 on Bim is dependent on Grx1′s thioltransferase activity. These findings indicate that Grx1 induction plays a key role in mediating the protective effect of laminar blood flow and suggest that Grx1 may be a potential therapeutic target for atherosclerosis.

## Introduction

It has been well recognized that oxidative stress is implicated in the pathogenesis of atherosclerosis^1,2^. The physiological feature of atherosclerosis at the early stage is the damage of endothelial cells, whose functions are critical for maintaining the integrity of vascular wall and homeostasis^3^. Oxidative stress elicited by the production of deleterious free radicals, either from disturbed blood flow (d-flow) or from exogenous factors such as smoking, may contribute to vascular endothelial malfunction and cell death^4,5^.

Apoptosis is a programmed cell suicide mechanism that plays a critical role in eliminating damaged cells and maintaining tissue homeostasis^6,7^. Apoptosis is generally regulated by two major families of proteins including Bcl-2 family^8–10^ and caspases^11,12^. The proapoptotic protein Bim belongs to Bcl-2 family and Bim expression is controlled by several transcription factors such as FoxO1. Phosphorylation of FoxO1 induced by Akt results in FoxO1 degradation and prevents Bim expression^13^. As an upstream factor of Bax, Bim may promote Bax activation^14–16^ and its localization at the outer mitochondrial membrane and form oligomers, which may further mediate mitochondrial apoptotic pathways, such as inducing the release of cytochrome c, a second mitochondria-derived activator of caspase/diablo homolog (Smac/DIABLO) and apoptosis inducing factor^17–19^. In addition, the stability of Bim can be regulated by c-Jun N-terminal kinase (JNK) which is a key enzyme responsive to stress stimuli. For instance, UV radiation^20^, γ irradiation^21^ or tumor necrosis factor (TNF)-related apoptosis-inducing ligand (TRAIL)^22^, can trigger JNK-dependent phosphorylation of Bim and subsequently activate mitochondrial apoptotic machinery. Although oxidative stress may also modulate JNK signaling^23^, it remains unknown whether Bim is involved in oxidative stress-induced apoptosis of endothelial cells.

In contrast to cell suicide, there are also intrinsic defense mechanisms in endothelial cells to actively cope with oxidative stress. Steady laminar blood flow (s-flow) was suggested to induce a reducing intracellular environment and thus protect endothelial cells from oxidative stress^5^. In our previous study, we found that short-term s-flow significantly increases the activity of glutaredoxin 1 (Grx1) which is required for Akt activation in endothelial cells^24^. Grx1 is a small dithiol protein with a conserved CPYC sequence in its active site^25,26^. It regulates intracellular redox balance through a disulfide exchange between the cysteinyl thiols in Grx1 and glutathione (GSH). As protein S-glutathionylation is emerging as a potential contributing factor in atherosclerosis^27^, it is conceivable that Grx1-mediated de-glutathionylation of proteins may play a role in regulating the pathogenesis of atherosclerosis. Moreover, Grx1 was shown to be an anti-apoptotic factor in rat cardiac H9c2 cells^28^.

Based on these previous findings, we hypothesized that Grx1 mediates the protective effect of steady laminar flow on endothelial cells against oxidative stress-induced apoptosis via inhibiting Bim. To test this hypothesis, we first compared the Grx1 and Bim expression in different flow regions by immunofluorescence staining. Our data show that the abundance of Grx1 is inversely correlated with the abundance of Bim in mouse aortic endothelial cells subjected to s-flow or d-flow. Further *in vitro* studies indicate that physiological s-flow (shear stress = 12 dyn/cm^2^) significantly inhibits Bim expression via deactivating its transcription factor FoxO1, which is regulated by Grx1-Akt signaling. In contrast, oxidative stress induced by H_2_O_2_ significantly inhibits Grx1 activity, increases the abundance of phosphorylated JNK (p-JNK), and promotes Bim stability and activation. Overexpression of Grx1 or pretreatment of s-flow attenuates H_2_O_2_-induced activation of JNK/Bim and apoptosis of endothelial cells. These findings suggest that s-flow increases Grx1 expression and helps endothelial cells combat oxidative stress via inhibiting Bim expression and activation.

## Results

### Different expression profiles of Grx1 and Bim in s-flow and d-flow regions

**Figure 1:**
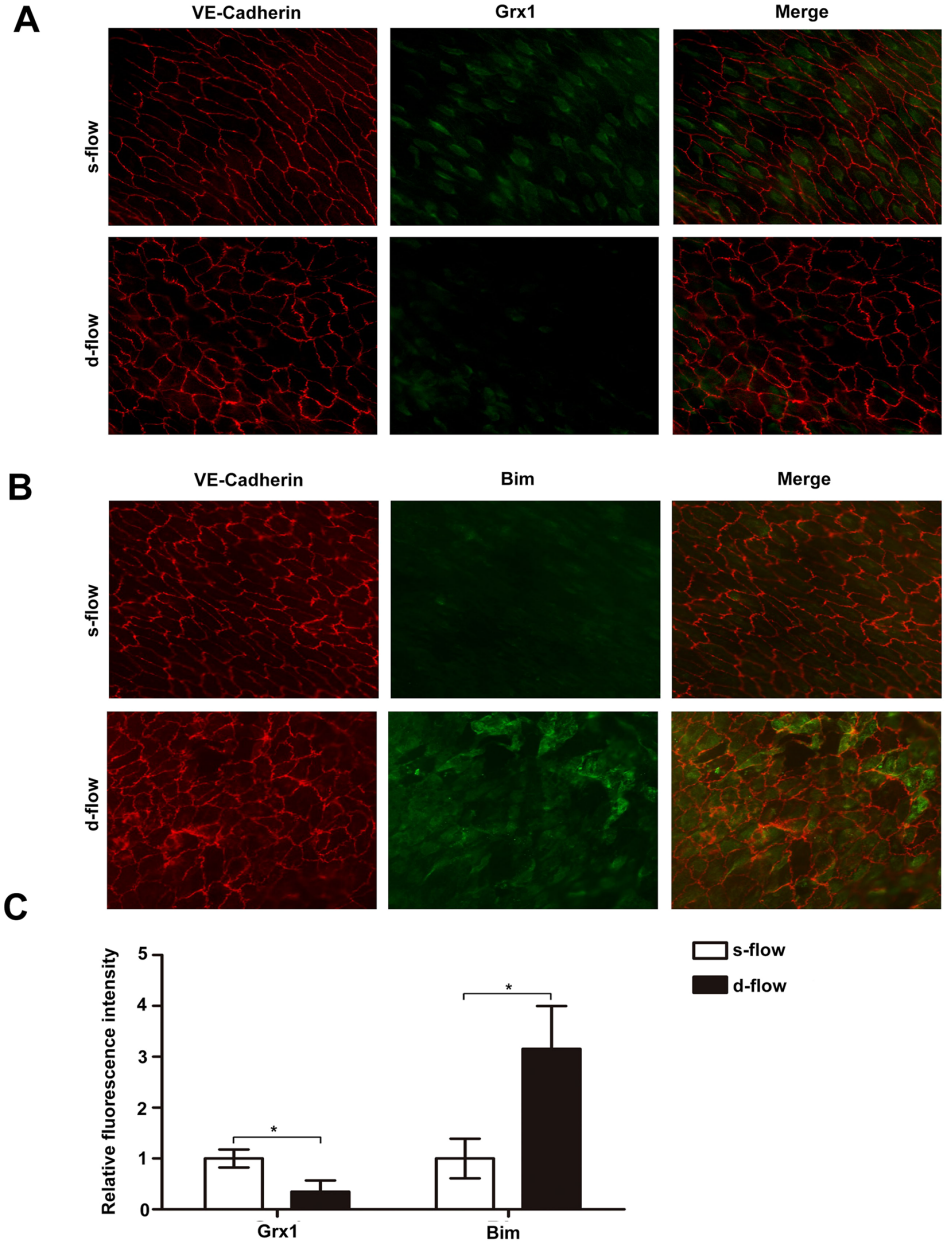
Different expression profiles of Grx1 and Bim in s-flow and d-flow regions. (**A**) The expression of Grx1 and (**B**) Bim was separately detected by enface immunofluorescence staining in mouse aorta regions subjected to s-flow or d-flow. The morphology of endothelial cells was visualized by VE-cadherin staining. (**C**) Three equal sections were selected from each slide. The fluorescence intensity of Grx1 and Bim under d-flow/s-flow was measured by ImageJ. Data are presented as the mean ± SD (*n* = 3); *, d-flow vs. s-flow, *p* < 0.05.

A growing body of evidence suggests that the local hemodynamic profile (s-flow or d-flow) may regulate the redox state and determine the function and fate of the endothelial cells. For instance, excess cell turnover in the regions of d-flow with low shear stress generates spatial difference in the endothelium^29^. By contrast, s-flow can protect endothelial cells from apoptosis by mediating the redox state of many ROS sensitive enzymes^30,31^. According to previous findings, we hypothesized that Grx1 and Bim have differential expression under s-flow and d-flow. Therefore, we examined the protein levels of Grx1 and Bim in different mouse aorta regions subjected to s-flow or d-flow using enface immunofluorescence staining. As shown in Fig. 1A, Grx1 expression is significantly increased in s-flow regions compared with d-flow regions. Conversely, the levels of Bim are remarkably reduced by s-flow, as compared with d-flow (Fig. 1B). Regardless of the types of blood flow (s-flow or d-flow), the levels of Grx1 are inversely correlated with the levels of Bim.

### Grx1 mediates the inhibitory effect of s-flow on Bim expression through regulating Akt-FoxO1

**Figure 2:**
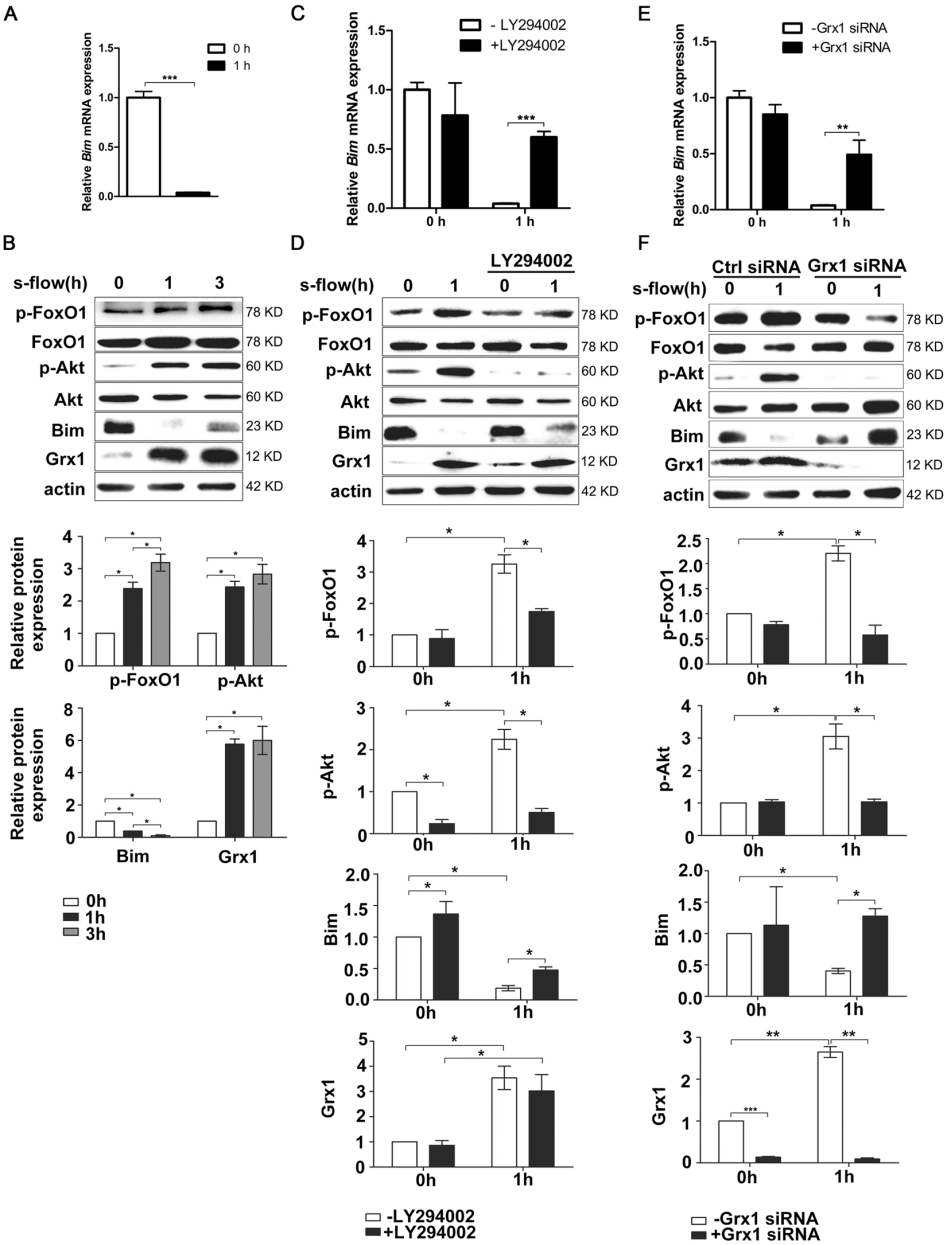
Grx1 mediates the inhibitory effect of s-flow on Bim expression through regulating Akt-FoxO1. (**A**,**B**) BAECs were exposed to s-flow for indicated time, and then analyzed by qPCR and Western blot using indicated antibodies. (**C**,**D**) BAECs were pretreated with Akt inhibitor LY294002 at 20 µM, and then exposed to s-flow for 1 h. The expression of indicated proteins was analyzed by qPCR and Western blot. (**E**,**F**) HUVEC were transfected with Grx1 siRNA then exposed to s-flow, the cell lysates were subjected to RT-PCR and immunoblots with indicated antibodies. *p < 0.05; **p < 0.01; ***p < 0.001.

Since Grx1 and Bim expression were altered by s-flow in an opposite manner, we hypothesized that Grx1 may mediate flow protection on endothelial cells by inhibiting Bim expression. To test this hypothesis, the expression of Bim and Grx1 was examined in endothelial cells exposed to s-flow *in vitro*. Consisting with the data from intact aortas in Fig. 1, s-flow significantly inhibited Bim expression on both mRNA level and protein level (Fig. 2A, 2B). Also, s-flow induces Grx1 expression *in vitro*.

Our previous study reported that Grx1 mediates s-flow-induced Akt activation^24^, and consequently phosphorylates FoxO1 (a member of the forkhead family of transcription factors), which can activate Bim expression. Therefore we hypothesized that Grx1 inhibits Bim expression by activating Akt/FoxO1 signaling. Indeed, we found that phosphorylation of both Akt and FoxO1 is significantly up-regulated by s-flow in endothelial cells. In contrast, pretreatment of Akt inhibitor LY294002 attenuated the inhibitory effect of s-flow on Bim expression in both mRNA and protein level (Fig. 2C, 2D). Grx1-specific siRNA abolished the effect of s-flow on Bim expression in both mRNA and protein level (Fig. 2E). This effect was reproduced by inhibiting Akt/FoxO1 phosphorylation (Fig. 2F). These results suggest that Grx1 is a critical regulator in s-flow-mediated inhibition of Bim expression by regulating Akt and FoxO1.

### Grx1 attenuates H_2_O_2_-induced Bim activation by regulating JNK phosphorylation

**Figure 3:**
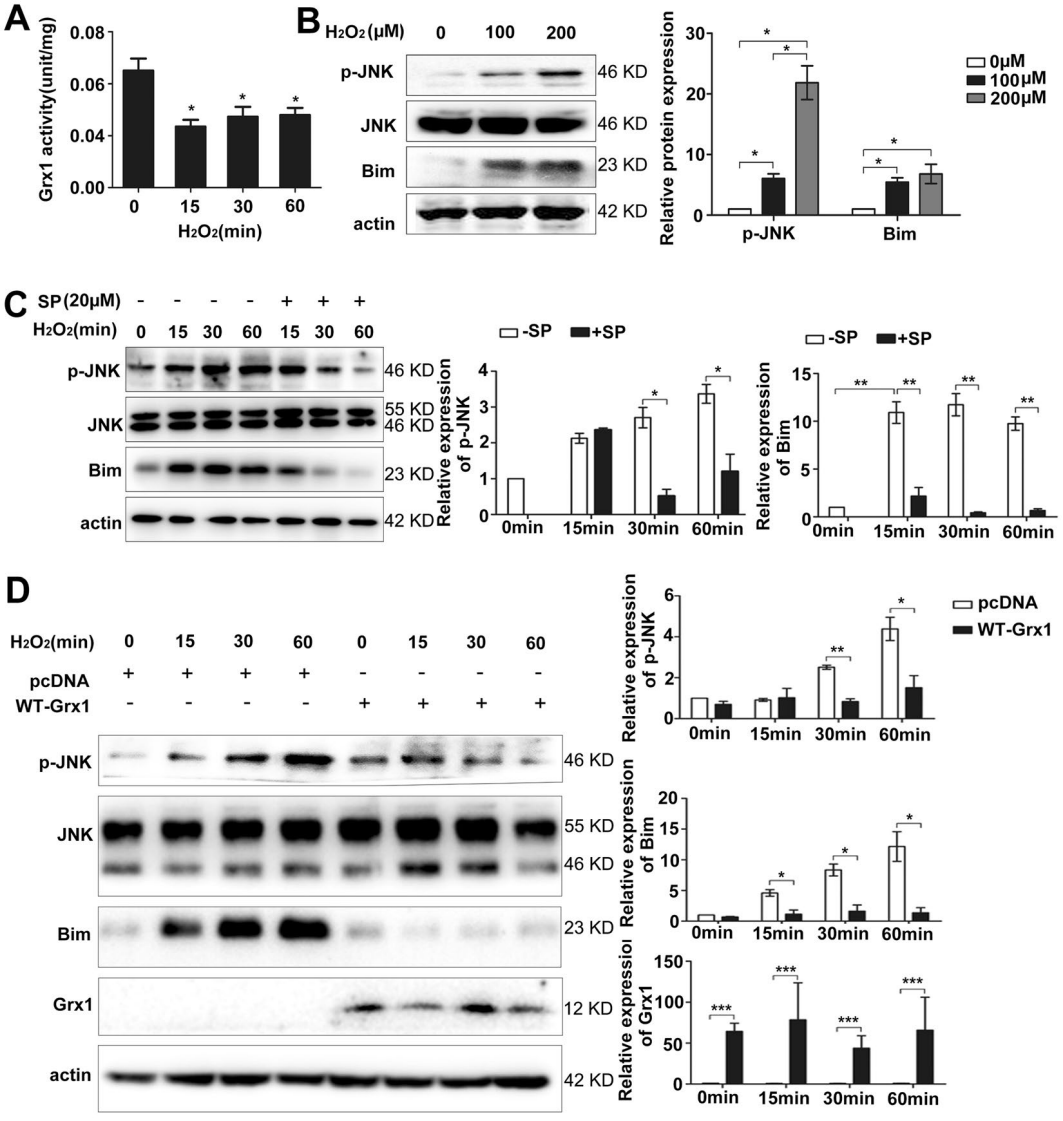
Grx1 attenuates H_2_O_2_-induced Bim activation by regulating JNK phosphorylation. (**A**) Equal amounts of total proteins were used to measure Grx1 activity. Grx1 activity was measured by Grx1 activity assay. One unit of Grx1 activity was defined as 1 µmol of NADPH oxidized per min under the standard assay conditions (data were expressed as mean ± SEM, *n* = 3). (**B**) BAECs were incubated with indicated doses of H_2_O_2_ for 60 min. p-JNK and Bim expression was analyzed by Western blots. (**C**) BAECs were pretreated with SP at 20 μM before incubated with H_2_O_2_ (300 µM) for the indicated time, and then subjected to SDS-PAGE. The protein levels of p-JNK, JNK and Bim were determined by Western blots. (**D**) BAECs were transfected with pcDNA3 or WT Grx1 and incubated with indicated doses of H_2_O_2_ for 60 min. The protein levels of p-JNK and Bim were determined by Western blots. *p < 0.05; **p < 0.01; ***p < 0.001.

Besides regulation at transcription level, the pro-apoptotic protein Bim can also be regulated at post-translational level. It has been previously shown that JNK-dependent phosphorylation of Bim enhances its proapoptotic activity through regulating its protein stability^32^. Therefore, we hypothesized that Grx1 regulates Bim activation through altering JNK phosphorylation. Grx1 activity is regulated by the redox states since only the reduced form of Grx1 is active. Oxidized Grx1 can be selectively recycled to the reduced form by GSH^33^. We used hydrogen peroxide to mimic the oxidative stress and examined Grx1’s activity after H_2_O_2_ treatment. Endothelial cells were exposed to H_2_O_2_ from 0 to 60 min, and Grx1 activity was assayed using whole cell lysates. As shown in Fig. 3A, H_2_O_2_ stably inhibits Grx1 activity as compared with the control, suggesting that H_2_O_2_ may inhibit Grx1 activity by maintaining Grx1 in the oxidized form.

We next examined the activation of JNK and Bim in response to H_2_O_2_. Endothelial cells were incubated with different concentrations of H_2_O_2_ for 60 min. As shown in Fig. 3B, H_2_O_2_ at 100 µM or 300 µM up-regulates the levels of phosphorylated JNK (p-JNK, an active form of JNK) without altering the total protein levels of JNK. Similarly, the expression of Bim is also enhanced by 100 µM and 300 µM H_2_O_2_, respectively. Time course experiments show that 300 µM H_2_O_2_ constantly increases the levels of p-JNK and Bim up to 60 min (Fig. 3C). To investigate whether H_2_O_2_-induced Bim activation and stability are JNK-dependent, endothelial cells were pretreated with 20 μM of SP600125 (SP), a specific inhibitor of JNK, and then exposed to H_2_O_2_ (300 µM) for up to 60 min. As shown in Fig. 3C, H_2_O_2_-induced phosphorylation of JNK and Bim expression is partially inhibited by pretreatment of SP. These data suggest that H_2_O_2_ could induce Bim activation and stability, which is p-JNK-dependent.

To determine the effects of Grx1 on H_2_O_2_-induced JNK and Bim activation, we transiently transfected endothelial cells with a wild-type (WT) Grx1 construct. The expression of Grx1 protein was confirmed by Western blot analysis (Fig. 3D). Activation of JNK and Bim was also examined in Grx1-overexpressing endothelial cells treated with H_2_O_2_. As shown in Fig. 3D, H_2_O_2_ increases the levels of both p-JNK and Bim, whereas these effects are attenuated by Grx1 overexpression. These data suggest that Grx1 may be an important regulator of H_2_O_2_-induced JNK and Bim activation.

### Grx1 attenuates H_2_O_2_-induced cell apoptosis in a thioltransferase activity-dependent manner

**Figure 4:**
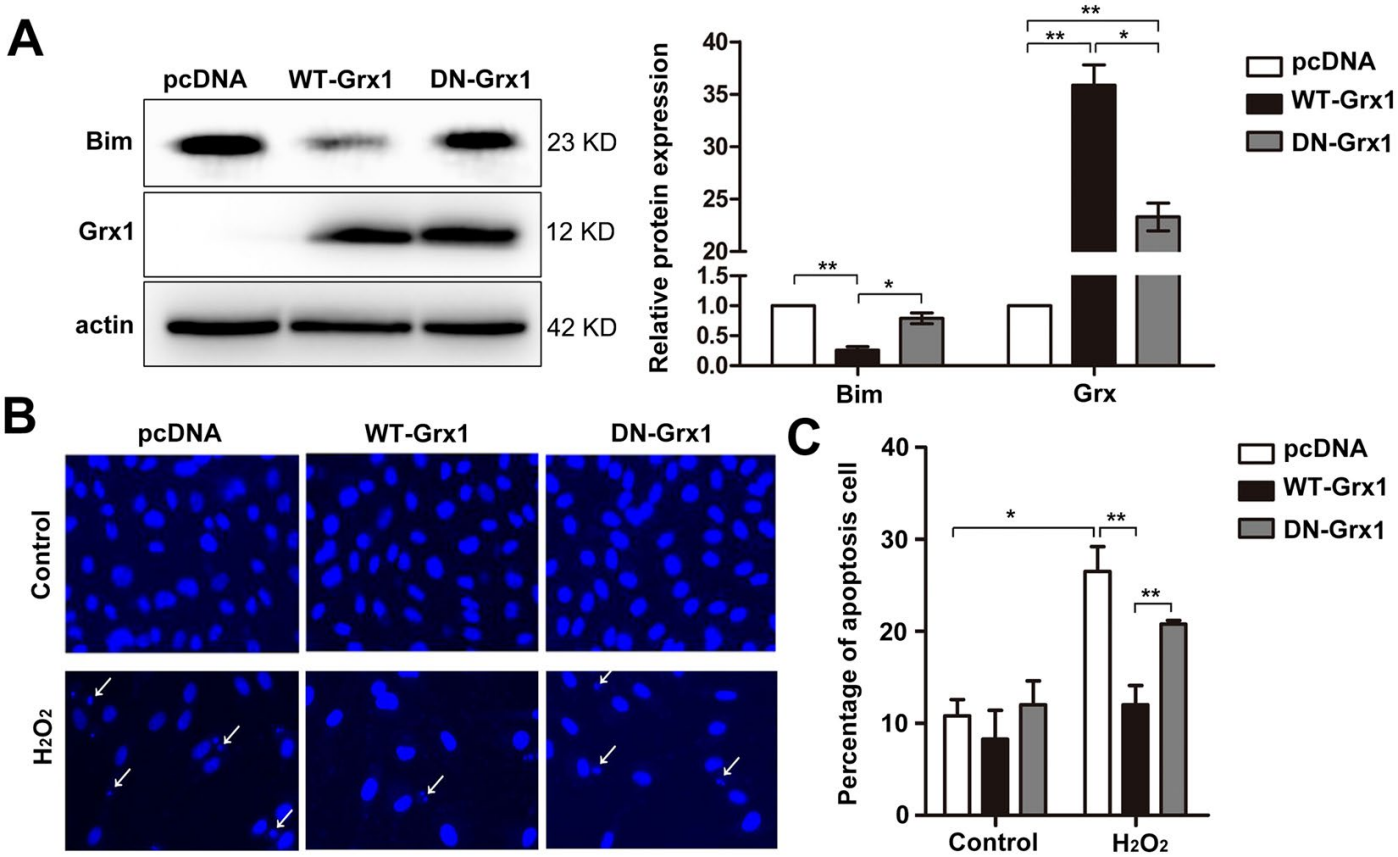
Grx1 inhibits H_2_O_2_-induced apoptosis which is dependent on Grx1 thioltransferase activity. (**A**) BAECs were transfected with pcDNA3, WT Grx1 or DN Grx1. Protein levels were determined by Western blots. (**B**) BAECs were transfected with pcDNA3, WT Grx1 or DN Grx1, followed by treatment with or without 300 μM of H_2_O_2_. After 24 h, cells were fixed, stained with DAPI, and apoptotic cells (indicated by white arrows) were counted under fluorescence microscopy. (**C**) BAECs cells were transfected with different constructs and then treated with or without H_2_O_2_. The apoptotic cells were examined by flow cytometry (TUNEL staining). The percentage of apoptotic cells in different groups was analyzed by Flowjo 7.6. Data are presented as the mean ± SD (*n* = 3); *p < 0.05; **p < 0.01.

Grx1 is a small (12-kDa) dithiol protein involved in many cellular events by regulating the redox status of cellular proteins. The catalytic domain of Grx1 protein contains a conserved CPYC sequence in which two cysteines at position 22 and 25 are essential for the thioltransferase activity of Grx1. To test whether the thioltransferase activity of Grx1 is required for the inhibition of Bim, WT Grx1 and C22/25 S mutated Grx1 (DN Grx1) were expressed in endothelial cells. As shown in Fig. 4A, Bim levels are considerably decreased in cells transiently transfected with WT Grx1, as compared to the control transfected with empty vector pcDNA3. Although the expression levels of DN Grx1 are comparable to those of WT Grx1, DN Grx1 lacking thioltransferase activity cannot inhibit the expression of Bim (Fig. 4A), suggesting that Grx1-mediated inhibition of Bim is dependent on its thioltransferase activity.

As Grx1 may modulate Bim expression and activation under oxidative stress, we next examined the effect of Grx1 on cell apoptosis by overexpressing WT Grx1 or DN Grx1 in endothelial cells. The transfected cells were either untreated (control), or treated with 300 µM of H_2_O_2._ After 24 h, cells were fixed, stained with 4′-6-diamidino-2-phenylindole (DAPI), and the pictures were taken under fluorescence microscopy. The apoptotic cells are indicated as the arrows shown (Fig. 4B). We found that the numbers of apoptotic cells are increased in all the cells treated with H_2_O_2_ compared to control. By contrast, WT Grx1, but not DN Grx1, significantly reduces H_2_O_2_-induced cell apoptosis (Fig. 4B). To further confirm these results, we used flow cytometry to count the apoptotic cells. The percentage of apoptotic cells is considerably decreased in cells transiently transfected with WT Grx1 (12.03% ± 2.09%) (Fig. 4C), as compared to empty vector pcDNA3 (26.50% ± 2.73%) and DN Grx1 (20.80% ± 0.37%). These results indicate that the anti-apoptotic effect of Grx1 in endothelial cells upon H_2_O_2_ exposure is thioltransferase activity-dependent.

### Grx1 mediates the protective effect of s-flow on H_2_O_2_-induced apoptosis by regulating activation of JNK and Bim

**Figure 5:**
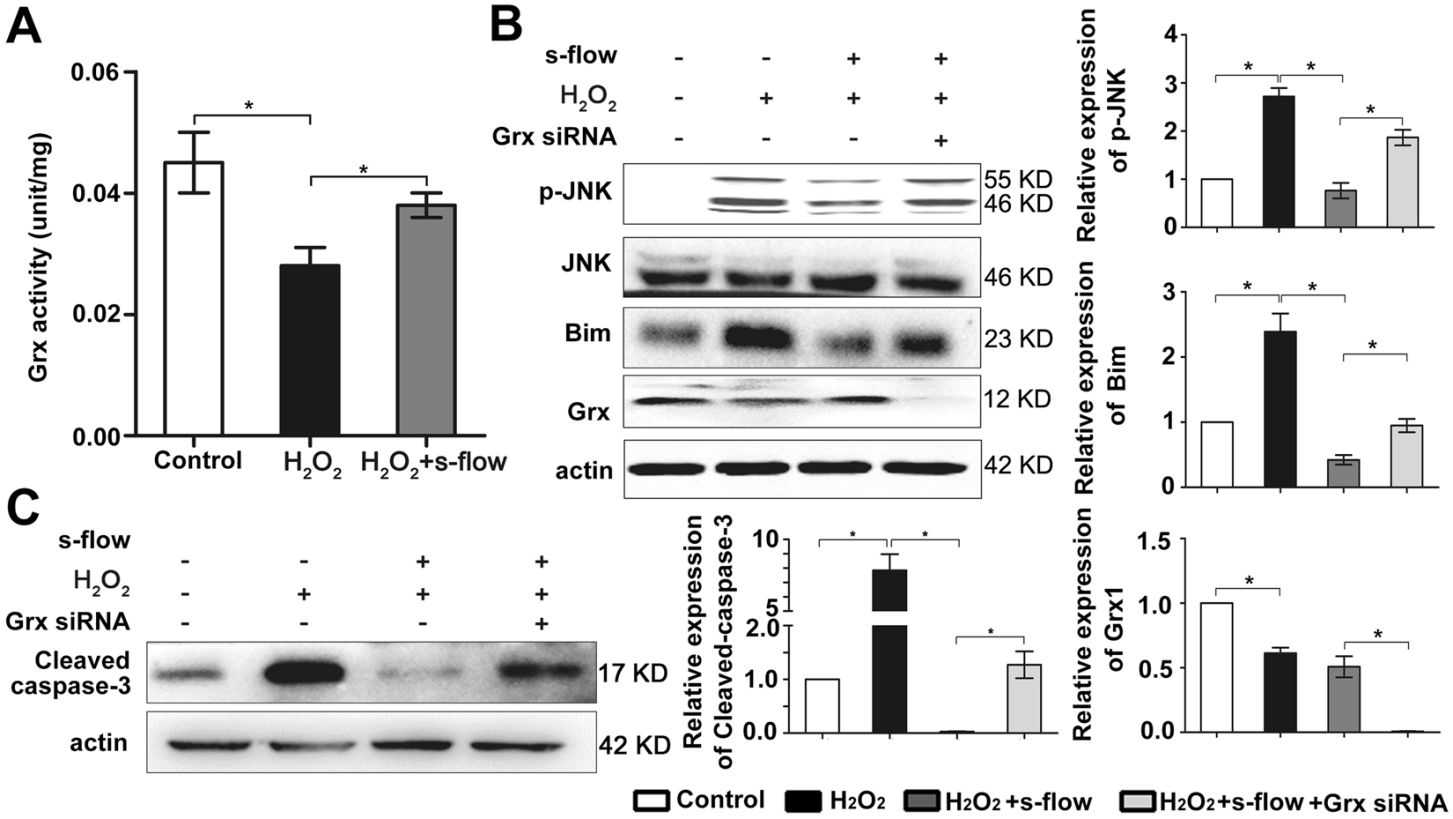
Grx1 mediates the effect of s-flow against apoptosis through inhibition of H_2_O_2_-induced JNK phosphorylation. (**A**) Equal amounts of total proteins were used to measure Grx1 activity. Grx1 activity was measured by Grx1 activity assay. One unit of Grx1 activity was defined as 1 µmol of NADPH oxidized per min under the standard assay conditions (data were expressed as mean ± SEM, *n* = 3). (**B**,**C**) Grx1 siRNA was transfected into HUVEC, which were subjected to s-flow before exposed to H_2_O_2_. After 1 h, cell lysates were subjected to SDS-PAGE and immunoblotting with the indicated antibodies. *p < 0.05.

As Grx1 reduces H_2_O_2_-induced apoptosis by inhibiting JNK/Bim activation, we thus hypothesized that Grx1 may mediate flow protection on endothelial cells by inhibiting JNK/Bim signaling. To test this hypothesis, we first examined the effect of s-flow on Grx1 activity after H_2_O_2_ treatment. As shown in Fig. 5A, while H_2_O_2_ inhibits Grx1 activity, s-flow prevents the inhibitory effect of H_2_O_2_ on Grx1. To test whether Grx1 mediates flow protection on endothelial cells by inhibiting JNK/Bim signaling, we transfected Grx1-specific siRNA into endothelial cells. After siRNA transfection, endothelial cells were pretreated with s-flow for 1 h before exposed to H_2_O_2_ (300 µM). Expression of p-JNK and Bim was analyzed by immunoblotting. As shown in Fig. 5B, s-flow attenuates H_2_O_2_-induced p-JNK and Bim. In contrast, knockdown of Grx1 abolishes the effects of s-flow on the p-JNK and Bim. It has been previously reported that the anti-apoptotic function of Grx1 under flow protection was reflected by the protein levels of cleaved caspase-3, which is the active form of caspase-3^34,35^. As shown in Fig. 5C, s-flow prevents H_2_O_2_ –induced caspase-3 activation. This protective effect of s-flow is attenuated when Grx1 is knocked down, indicating that Grx1 is required for s-flow-mediated inhibition of oxidative stress-induced cell death in endothelial cells.

## Discussion

**Figure 6:**
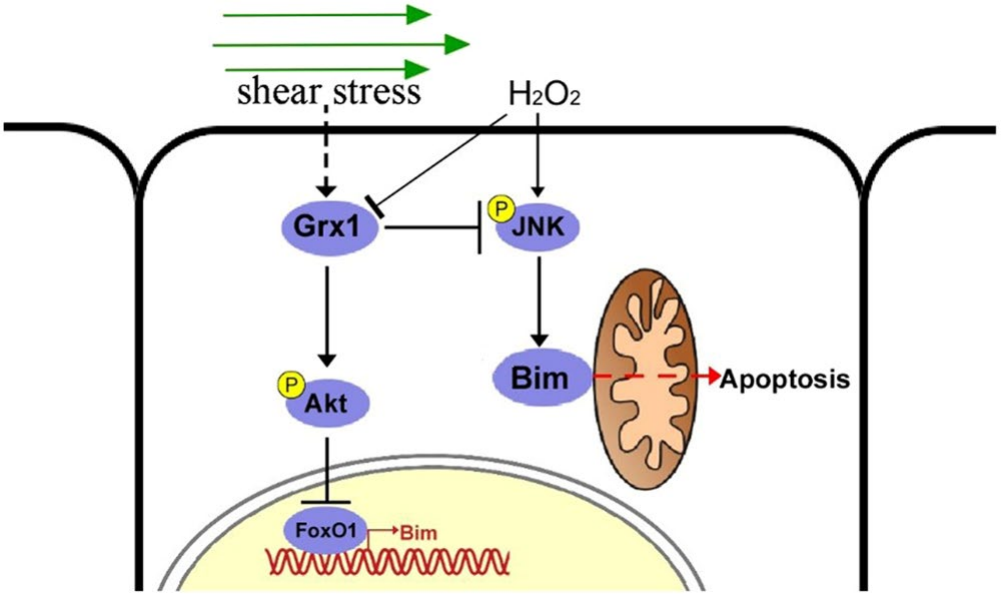
A proposed model of Grx1/Bim in modulating endothelial cell apoptosis. Under s-flow, Grx1 is up-regulated and thus attenuates H_2_O_2_-induced apoptosis of endothelial cells by suppressing Bim transcription via Akt/FoxO1, as well as inhibiting the activation of JNK/Bim.

Our present study reveals an important role of Grx1 in s-flow’s anti-apoptotic function against oxidative stress. The molecular mechanisms accounting for the protective effects of Grx1 and s-flow are summarized as followings: Grx1 is markedly up-regulated under the s-flow both *in vivo* and *in vitro*. Grx1 attenuates oxidative stress-induced apoptosis of endothelial cells by inhibiting pro-apoptotic protein Bim expression and activation. These protective effects of Grx1 depend on its thioltransferase activity. Based on our findings, we propose a novel mechanistic model (Fig. 6). This model is supported by following experimental evidence: (1) The expression levels of Grx1 are inversely correlated with the levels of Bim in s-flow regions (Fig. 1); (2) s-flow can induce the expression of Grx1 which inhibits Bim expression by regulating its transcription factor FoxO1 (Fig. 2); (3) Grx1 down-regulates H_2_O_2_-induced Bim activation by regulating phosphorylation of JNK (Fig. 3); (4) H_2_O_2_-induced endothelial cell apoptosis can be inhibited by Grx1 (Fig. 4); (5) Grx1 mediates the protective effect of s-flow against oxidative stress-induced apoptosis by regulating Bim (Fig. 5).

Our results from enface immunofluorescence staining provide strong evidence that s-flow activates Grx1 expression and inhibits Bim expression. Grx1 inhibits Bim expression through activating Akt and inducing phosphorylation of the downstream target FoxO1. Phosphorylated FoxO1 is ubiquitinated and degraded in the cytoplasm, subsequently decreasing Bim transcription. S-flow maintains endothelial function through promoting cell survival and preventing apoptosis^36,37^. Our previous study demonstrated that Grx1 is an important mediator for flow-induced Akt and eNOS activation, which maintains endothelial cell survival^24^. There may be pathways besides Akt involved. The present study further demonstrates the key role of Grx1 in the anti-apoptotic function of s-flow by inhibiting Bim. Taken together, our results show that Grx1 plays an essential role in the regulation of vascular homeostasis under s-flow.

We show that Grx1 inhibits JNK phosphorylation and Bim activation induced by H_2_O_2_. The JNK/Bim pathway is activated in response to the intracellular ROS. The protein levels and apoptotic function of Bim are regulated at different levels. Here we present that Bim is up-regulated by JNK in response to oxidative stress. However the interaction sites between Bim and JNK have not been determined. It is very likely that JNK-dependent phosphorylation of Bim is at Ser-65 and Thr-112, as previous studies suggested^38^. In nerve growth factor (NGF) -deprived sympathetic neurons, JNK-dependent phosphorylation of Bim increases its apoptotic ability through increasing its stability and facilitating the translocation of Bim to mitochondria^39^. This scenario needs to be validated in H_2_O_2_-treated endothelial cells. In addition, it has been reported that Grx may exert its anti-apoptotic effects via inhibiting NF-kB activation or FasL signaling^40,41^. Whether NF-kB and FasL pathways are involved in Grx-mediated protection of s-flow on EC apoptosis needs to be further investigated.

Our data demonstrate a critical inhibitory effect of Grx1 on H_2_O_2_-induced endothelial cell apoptosis. As one of the redox-sensing molecules, Grx1 can sense cellular oxidative stress through preventing JNK phosphorylation. But there are still gaps between Grx1 and JNK inactivation. Grx1 contains two redox-active cysteine residues, -Cys-Gly-Pro-Cys-. In a previous study with breast cancer cells, intracellular ROS induced the formation of an intramolecular disulfide bond in Grx1, which led to a conformational change^42^. This change resulted in the dissociation of Grx1 from apoptosis signal-regulating kinase 1 (ASK1), and subsequently activated ASK1. Activation of ASK1 induces the JNK/Bim pathway which eventually leads to cell death^42^. Our current study shows that Grx1 prevents the activation of JNK/Bim pathway in endothelial cells under shear stress. Therefore, it is possible that Grx1 may direct target ASK1 under shear stress. Furthermore, a recent study has shown that Grx1 can protect retinal pigment epithelial cells against H_2_O_2_-induced apoptosis^43^. Grx1 may promote Akt phosphorylation by preventing Akt glutathionylation.

After demonstrating Grx1 inhibits H_2_O_2_-induced cell apoptosis by suppressing the JNK/Bim pathway, we next determined the role of Grx1 in shear stress. The results indicate that s-flow can stimulate Grx1 expression and activity, and prevent H_2_O_2-_induced apoptosis by inhibiting p-JNK and Bim. This is consistent with our previous study that the activation of Grx1 by s-flow is through the regulation of Grx system^24^. Briefly, s-flow increases the activity of glutathione reductase (GR) and thus increases the ratio of GSH/GSSG. In the Grx system, electrons are transferred from NADPH to GR, GSH, and finally to Grx1. As Grx1 is only functional under the reduced form, Grx1 activity is increased by s-flow via maintaining a reducing intracellular environment in a GR-dependent manner^24^. In the branched region of the arteries, it is possible that d-flow lacks the ability to stimulate GR and thus Grx1 is in an inactive form. The mechanism we propose in the present study provides an insight into the athero-protective role of s-flow. It also gives one plausible explanation of why atherosclerosis lesions occur in disturbed flow regions in the artery.

In this report, our data indicate that s-flow protects endothelial cells through tuning the redox status and up-regulating Grx1 expression. Grx1 exerts potent anti-apoptotic effect through inhibiting Bim expression and activation. Given the fact that clinical intervention of hemodynamic profile of the blood flow is technically difficult, our study raises the possibility that Grx1 may be a promising therapeutic target for atherosclerosis.

## Materials and Methods

### DNA constructs and reagents

Grx1 expression plasmid and C22/25 S mutated Grx1 construct were prepared as previously described^24^. Grx1 siRNA was designed as previously described^44^ and ordered from Dharmacon. Control siRNA was ordered from Qiagen.

### Cell culture and flow experiments

Bovine aortic endothelial cells (BAECs) were isolated as previously described^45^, and cultured in medium 199 (M-199) (Gibco) supplemented with 100 U/mL of penicillin and 100 μg/mL of streptomycin (Gibco), 1% MEM amino acids (Gibco), 1% MEM vitamins (Cellgro), 10% fetal clone III (bovine serum product, HyClone), at 37 °C with 5% CO_2_. Cells at passages 5 to 8 were used for experiments. Human umbilical vein endothelial cells (HUVECs) were isolated and maintained in Medium 200 (Cascade Biologics) with low serum growth supplements. Cells at passages 2 to 4 were used. To reduce the background signals of kinases, cells were cultured for one day in serum free medium prior to the flow experiments (shear stress of 12 dyn/cm^2^) using a cone and plate viscometer. The cells were handled according to the regulations of the Institute of Basic Medical Sciences of the Chinese Academy of Medical Sciences, Beijing, China, and the study protocol was approved by the Institutional Review Board of the Institute of Basic Medical Sciences, the Chinese Academy of Medical Sciences. Written informed consent was obtained either from the donor or a close relative for HUVEC isolation.

### RNA isolation and RT-PCR

Trizol (Invitrogen) was used to extract total RNA from cells as the manufacture instructed. Nanodrop was used to measure the concentration and purity of the RNA. The first strand cDNA was obtained by reverse transcription using M-MLV (Invitrogen) from 1 μg of total RNA using oligo-dT as primer. The mRNA levels of Bim and was determined by qPCR. The primer sequencing for Bim: (F- CAAGGTAATCCTGAAGGCAA, R- CACTGAGATAGTGGTTGAAG) GAPDH was used as reference gene: (F- ACAACTTTGGTATCGTGGAAGG, R- GCCATCACGCCACAGTTTC).

### Transient transfection with DNA constructs

BAECs were cultured in a 60-mm dish for 24 h prior to transfection. Cells at 90% confluence were used for transfection. Lipofectamine 2000 reagent (Invitrogen) was used to transfect 2 μg DNA (pcDNA3 vector was used as a control) per dish as manufacturer’s instructions. After 4 h, the medium was replaced with the fresh medium containing 10% serum.

### Gene knockdown

HUVEC at 90% confluence were used for transfection with 100 nmol/L Grx1 or control siRNA and Lipofectamine 2000 in OptiMEM medium, according to manufacturer’s instructions (Invitrogen). After incubation for 2 h, the medium with 5% serum was added.

### Cell lysate preparation

Cells were harvested by ice-cold phosphate-buffered saline (PBS 150 mM; NaCl 20 mM; Na_2_PO_4_, pH 7.4) on ice, lysed with lysis buffer (150 mM NaCl, 1 mM EDTA, 1 mM EGTA, 1% Triton X-100, 2.5 mM sodium pyrophosphate, 5 mM NaF, 1 mM Na_3_VO_4_ plus 1:1000 protein inhibitor cocktail (PIC, Sigma), and collected by centrifugation (10000 rpm, 10 min). Bradford assay (Bio-Rad) was performed to determine the protein concentration.

### Western blot analysis

Total proteins were separated by SDS-PAGE and transferred to nitrocellulose membranes, followed by the incubation with appropriate antibodies: Grx1 (American Diagnostica,1:1000), FoxO1 (Cell Signaling, 1:1000), phospho-FoxO1 (Cell Signaling, 1:1000), Akt (Cell Signaling, 1:1000), phospho-Akt (Cell Signaling, 1:1000), Actin (Santa Cruz,1:1000), Bim (BD Bioscience,1:500), Cleaved caspase-3 (Cell Signaling, 1:1000), phospho-JNK (Cell Signaling,1:500), and JNK antibodies (Cell Signaling,1:500). Immunoreactive proteins were visualized by Kodak film development system after washing and incubating with secondary antibodies.

### Grx1 activity assay

Grx1 activity in BAEC lysates was examined by measuring the change in absorbance of NADPH at 340 nm, as previously described^24,46,47^. Briefly, Grx1 activity was determined by monitoring the decrease in absorbance of NADPH at 340 nm with Beckman DU 640 Spectrophotometer. All reagents for the assay were ordered from Sigma.

### Cell apoptosis assay

Flow cytometric analysis was applied using BAEC cells with Annexin V-FITC/ propidium iodide (PI) Apoptosis Detection Kit (KeyGEN Biotech, Nanjing, China) according to the manufacturer’s instructions. Briefly, after treatment with various concentrations of H_2_O_2_, cells were harvested and washed with ice-cold PBS twice, and then resuspended in binding buffer at a concentration of 1 × 10^6^ cells/mL. Then, 5 µL of annexin V-FITC and 5 µL of PI were added. The cells were incubated for 15 min in the dark and the quantification of apoptosis was analyzed using flow cytometry (Becton Dickinson, Franklin Lakes, NJ, USA). The acquisition and analysis were performed using MoFlow (Beckman Coulter, Atlanta, GA, USA).

### En Face immunofluorescence staining

Immunofluorescence staining of mouse aortic endothelial cells was performed using 12-week-old C57BL/6 mice as previously described^48^ with several modifications. Briefly, mice were anesthetized with ketamine/xylazine cocktail (0.13/0.0088 mg/g body weight). Then the jugular veins were cut and the arterial tree was perfused from left ventricle with saline containing 40 USPU/mL heparin for 5 min, followed by pre-chilled 4% paraformaldehyde in PBS (pH 7.4) perfusion for 10 min. Subsequently, we dissected the whole aorta from iliac bifurcation to the heart. The aorta was cut open longitudinally, permeabilized with 0.1% Triton X-100 in PBS for 10 min and blocked with 10% normal goat serum in Tris-buffered saline (TBS) containing 2.5% Tween-20 for 1 h at room temperature. Next, we incubated the aortas with 5 µg/mL rabbit anti-Grx1 or 2 µg/mL rabbit anti-Bim, and 5 µg/mL rat anti-VE-Cadherin in the blocking buffer overnight at 4 °C. After washing with TBS containing 2.5% Tween-20 for three times, fluorescence-conjugated secondary antibodies (1:1000 dilution, Alexa Fluor 546 labeled anti-rabbit IgG and Alexa Fluor 488 labeled anti-rat IgG, respectively) were applied for 1 h at room temperature. Finally, we mounted aortas in the ProLong antifade reagent (Invitrogen, Eugene, OR) after washing t times. We examined aortas using a laser-scanning confocal microscope (FV-1000 mounted on IX81, Olympus) with UPlanSApo 20X or UplanFL N 40X lens. The quantification of Grx1/Bim by image analysis was visualized in Image-Pro. All animal experiments were performed in accordance with guidelines issued by the Committee on Animal Research of Peking Union Medical College and were approved by the institutional Review Board of Institute of Basic Medical Sciences, Chinese Academy of Medical Sciences.

### Statistical analysis

The experiments were repeated at least three times. All the data are expressed as mean ± SEM. A two-tailed Student’s t-test was applied to compare protein expression, enzyme activity and apoptotic cells.

### Data availability

All data generated or analysed during this study are included in this published article.

## Electronic supplementary material

Supplementary Information - gel image
