# Supplementary material for: Glutaredoxin 1 mediates the protective effect of steady laminar flow on endothelial cells against oxidative stress-induced apoptosis via inhibiting Bim
Source: Sci Rep. 2017 Nov 14;7:15539. doi: 10.1038/s41598-017-15672-3 (PMC5686153; doi:10.1038/s41598-017-15672-3)

Glutaredoxin 1 mediates the protective effect of steady laminar flow on endothelial cells against oxidative stress-induced apoptosis via inhibiting Bim

Yao Li[a,1], Meng Ren[a,1], Xiaoqun Wang[b,1], Xingxing Cui[a], Hongmei Zhao[a], Chuanrong Zhao[c], Jing Zhou[c], Yanan Guo[d], Yi Hu[e,*], Chen Yan[d], Bradford Berk[d], Jing Wang[a,*]

[a] *State Key Laboratory of Medical Molecular Biology, Institute of Basic Medical Sciences, Chinese Academy of Medical Sciences, Department of Pathophysiology, Peking Union Medical College, Tsinghua University, Beijing 100005, China*

[b] *Department of Cardiology, Ruijin Hospital, Shanghai Jiao-Tong University school of medicine, Shanghai, 200025, China*

[c] *Department of Physiology and Pathophysiology, School of Basic Medical Sciences, Peking University, Beijing, China; Key Laboratory of Molecular Cardiovascular Science, Ministry of Education, Beijing, China*

[d] *Aab Cardiovascular Research Institute, School of Medicine and Dentistry, University of Rochester, Rochester, NY 14642, USA*

[e] *CAS Key Laboratory for Biomedical Effects of Nanomaterials and Nanosafety, Multi-disciplinary Research Division, Institute of High Energy Physics, Chinese Academy of Sciences (CAS), Beijing 100049, China*

[1] These authors contributed equally to this work.

*Corresponding author

E-mail addresses: huyi@ihep.ac.cn (Y. Hu), wangjing@ibms.pumc.edu.cn (J. Wang).

Figure 2A

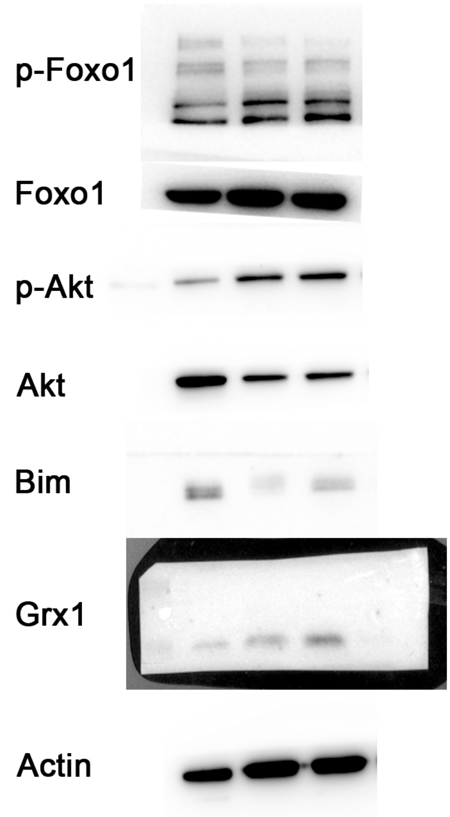

Figure 2B

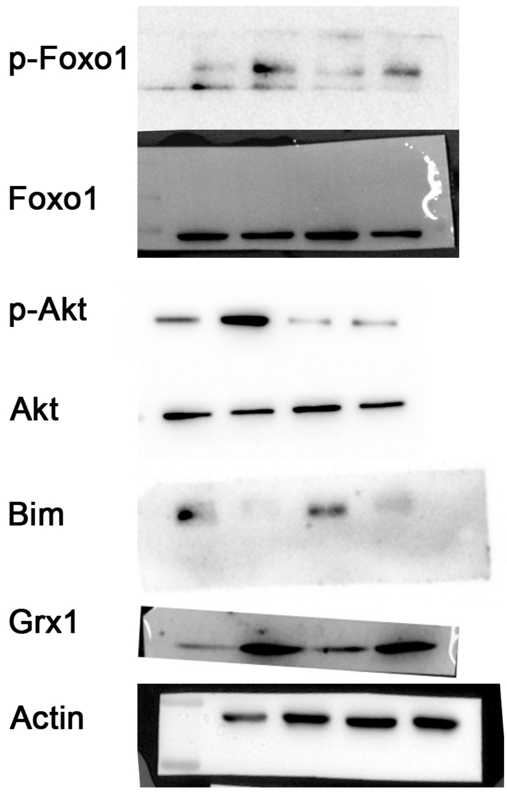

Figure 2C

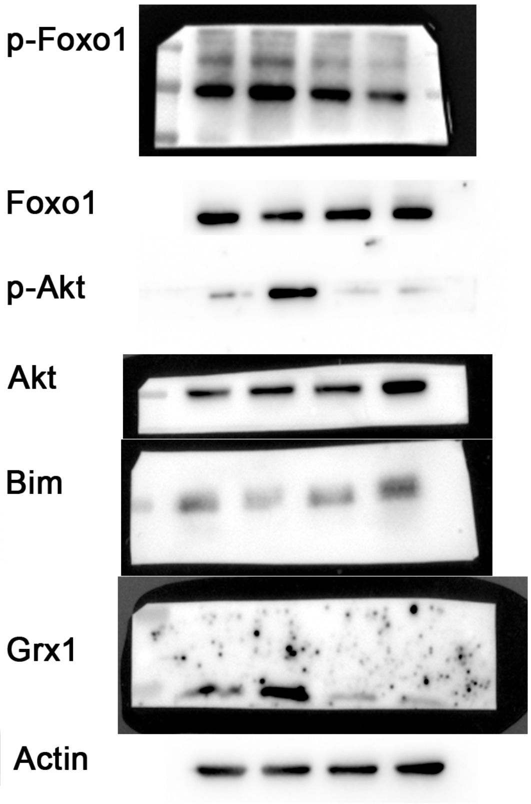

Figure 3B

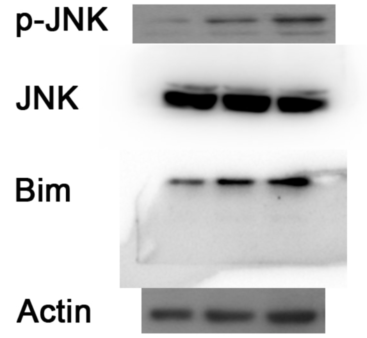

Figure 3C

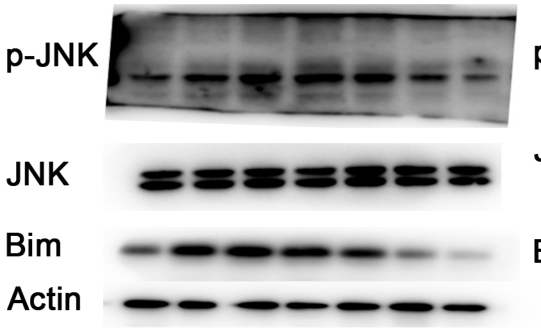

Figure 3D

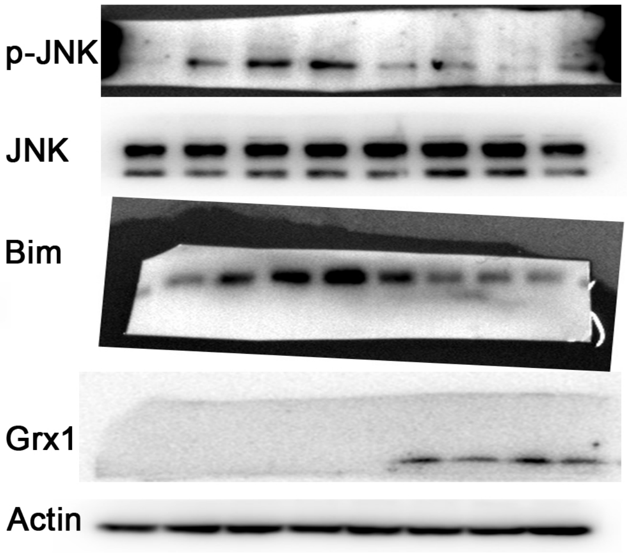

Figure 4A

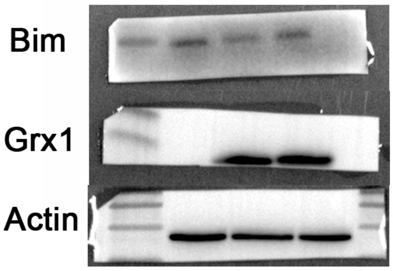

Figure 5B

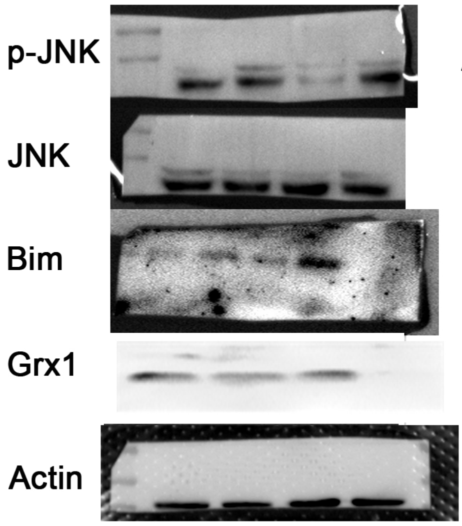

Figure 5C

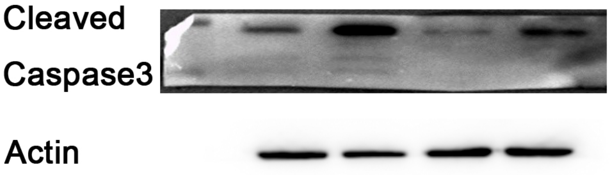

Supplement: Supplementary file 1 — Supplementary Information - gel image [file 41598_2017_15672_MOESM1_ESM.pdf]
